# Unveiling the Native Morphology of Extracellular Vesicles from Human Cerebrospinal Fluid by Atomic Force and Cryogenic Electron Microscopy

**DOI:** 10.3390/biomedicines10061251

**Published:** 2022-05-27

**Authors:** Mario Kurtjak, Sami Kereïche, Damir Klepac, Hrvoje Križan, Marko Perčić, Vedrana Krušić Alić, Teja Lavrin, Metka Lenassi, Karmen Wechtersbach, Nika Kojc, Marija Vukomanović, Siniša Zrna, Maša Biberić, Robert Domitrović, Kristina Grabušić, Mladenka Malenica

**Affiliations:** 1 Advanced Materials Department, Jožef Stefan Institute, SI-1000 Ljubljana, Slovenia; mario.kurtjak@ijs.si (M.K.); marija.vukomanovic@ijs.si (M.V.); 2 Institute of Biology and Medical Genetics, First Faculty of Medicine, Charles University, CZ-12800 Prague, Czech Republic; sami.kereiche@lf1.cuni.cz; 3 Department of Medical Chemistry, Biochemistry and Clinical Chemistry, Faculty of Medicine, University of Rijeka, HR-51000 Rijeka, Croatia; damir.klepac@medri.uniri.hr (D.K.); hrvoje.krizan@medri.uniri.hr (H.K.); robert.domitrovic@medri.uniri.hr (R.D.); 4 Centre for Micro- and Nanosciences and Technologies, University of Rijeka, HR-51000 Rijeka, Croatia; mpercic@riteh.hr; 5 Faculty of Engineering, University of Rijeka, HR-51000 Rijeka, Croatia; 6 Department of Physiology, Immunology and Pathophysiology, Faculty of Medicine, University of Rijeka, HR-51000 Rijeka, Croatia; vedrana.krusic@medri.uniri.hr (V.K.A.); kristina.grabusic@medri.uniri.hr (K.G.); 7 Institute of Biochemistry and Molecular Genetics, Faculty of Medicine, University of Ljubljana, SI-1000 Ljubljana, Slovenia; teja.lavrin@mf.uni-lj.si (T.L.); metka.lenassi@mf.uni-lj.si (M.L.); 8 Institute of Pathology, Faculty of Medicine, University of Ljubljana, SI-1000 Ljubljana, Slovenia; karmen.wechtersbach@mf.uni-lj.si (K.W.); nika.kojc@mf.uni-lj.si (N.K.); 9 Pula General Hospital, HR-52100 Pula, Croatia; sinisa.zrna@gmail.com (S.Z.); masa.biberic@gmail.com (M.B.)

**Keywords:** extracellular vesicles, cerebrospinal fluid, size-exclusion chromatography, atomic force microscopy, cryogenic transmission electron microscopy, morphology

## Abstract

Extracellular vesicles (EVs) are membranous structures in biofluids with enormous diagnostic/prognostic potential for application in liquid biopsies. Any such downstream application requires a detailed characterization of EV concentration, size and morphology. This study aimed to observe the native morphology of EVs in human cerebrospinal fluid after traumatic brain injury. Therefore, they were separated by gravity-driven size-exclusion chromatography (SEC) and investigated by atomic force microscopy (AFM) in liquid and cryogenic transmission electron microscopy (cryo-TEM). The enrichment of EVs in early SEC fractions was confirmed by immunoblot for transmembrane proteins CD9 and CD81. These fractions were then pooled, and the concentration and particle size distribution were determined by Tunable Resistive Pulse Sensing (around 10^10^ particles/mL, mode 100 nm) and Nanoparticle Tracking Analysis (around 10^9^ particles/mL, mode 150 nm). Liquid AFM and cryo-TEM investigations showed mode sizes of about 60 and 90 nm, respectively, and various morphology features. AFM revealed round, concave, multilobed EV structures; and cryo-TEM identified single, double and multi-membrane EVs. By combining AFM for the surface morphology investigation and cryo-TEM for internal structure differentiation, EV morphological subpopulations in cerebrospinal fluid could be identified. These subpopulations should be further investigated because they could have different biological functions.

## 1. Introduction

Extracellular vesicles (EVs) are a heterogeneous group of nano-sized particles in biofluids composed of nucleic acids, proteins, peptides and lipids and enclosed by a lipid membrane containing proteins, e.g., tetraspanins, receptors and other molecules [1]. EVs from the cerebrospinal fluid (CSF) are implicated in diverse physiological processes of the brain and can be used as biomarkers for brain disorders. Furthermore, EVs possess therapeutic potential, since they can cross the blood–brain barrier while carrying bioactive cargo that can influence the molecular mechanisms of the recipient cell. This is significant for organs that are not readily accessible, such as the brain [2,3].

Based on their biogenesis or release pathways, we can classify EVs mainly as microvesicles (~100 nm to 1 µm in size), formed by outward budding of the cell membrane, and widely investigated exosomes (~30–150 nm), which are formed by inward budding of endosomes, leading to multivesicular bodies that fuse with the plasma membrane. Exosomes are detected by protein markers, e.g., CD9, CD63, CD81 and others. Proteomic studies showed heterogeneity in the protein cargo, indicating additionally the existence of many subclassifications of EVs [4].

Before conducting any liquid biopsy studies involving EVs, a detailed characterization of their concentration, size and morphology is needed. Furthermore, their subpopulations have to be identified. The first step involves removal of the soluble proteins and other contaminants that could interfere with the results [5]. There have been numerous investigations comparing different conventionally applied separation methods: ultracentrifugation (UC), size-based separation (size exclusion chromatography (SEC) and ultrafiltration), polymer-based precipitation and immunoaffinity-based precipitation. They have been recently evaluated on human plasma and CSF by Ter-Ovanesyan et al. [6]. Moreover, new methods are emerging, e.g., microfluidic devices [7,8], clog-free ultrafiltration (EXODUS) [9] and nanomaterial-based isolations [10]. Each method has its advantages and disadvantages and needs to be optimized according to the biofluid and the research hypothesis. In this research, we chose gravity-driven SEC because of: (i) mild separation conditions in comparison with other commonly used methods to best preserve the native morphology of EVs; (ii) effective elimination of contaminants (albumin and immunoglobulin, the two most abundant proteins in CSF), which can hinder identification of low-abundance proteins [5]. Nevertheless, two limitations of SEC separation of EVs should be pointed out: (i) its inability to separate EVs from other particles of similar size, including lipoproteins [11], and (ii) dilution of the sample, which can influence the level of protein detection, especially since the initial particle concentration in CSF is lower than in other biofluids (e.g., blood and plasma). Researchers aim to overcome the dilution problem by various enrichment methods [12,13].

After separation, several methods are usually applied for EV quantification (size distribution and concentration measurements), such as Tunable Resistive Pulse Sensing (TRPS), which calculates the volume of the EVs from a change in electrical resistance caused by a blockage of particles passing through a nanopore membrane; Nanoparticle Tracking Analysis (NTA) measuring Brownian motion through a series of digital camera images of the trajectories of individual scattering objects and their displacements, related to each object’s size; and Dynamic Light Scattering (DLS), which relies on fluctuations of the scattered light intensity due to Brownian motion [14,15,16]. NTA and TRPS have been applied for quantification of EVs isolated by UC from the CSF of brain tumor patients, patients treated for hydrocephalus and those with no known comorbidities [5,12,17]. Additionally, NTA was used to monitor the kinetic changes in size and concentration of particles in the native CSF from patients after traumatic brain injury (TBI) [18].

Further on, microscopy methods are applied for EV morphology visualization, as recommended by the International Society for Extracellular Vesicles (ISEV), to assess sample heterogeneity and EV morphological properties [19,20]. Sample preparation for microscopy that requires dry samples can destroy the structure of EVs, e.g., simple drying in the air forces a “cup shape” due to the high surface tension of water. Then, an electron beam can damage the sample and the positive/negative staining to achieve better contrast in TEM can introduce artefacts in the form of electron-dense precipitates [21,22]. A beam can also interfere with the detection of the proteins on the surface of an EV when applied for distinction of subpopulations. Therefore, preservation of the surface for immuno-based morphological detection is required.

Atomic force microscopy (AFM) in liquid and cryogenic transmission electron microscopy (cryo-TEM) importantly preserve near-native morphology. AFM in liquid enables calculation of particle size distribution and concentration, 3D visualization and topography examination. It can also simultaneously measure biomechanical properties [23], and is a less expensive and more convenient option than cryo-TEM. Yet, cryo-TEM can provide additional and more detailed information about the shape, structure, morphology and topography, so it is best to use both methods for subpopulation studies.

The aim of this study was to evaluate the native morphology of chromatographically isolated EVs from human CSF after TBI by applying AFM in liquid and cryo-TEM. We quantified the concentration and size distribution of particles after SEC by NTA and TRPS and validated the quantification with microscopy methods. Furthermore, the information about the shape, structure, morphology and topography of EVs obtained by AFM is compared to the cryo-TEM images for internal structure differentiation to improve understanding of the EV subpopulations in the human CSF. 

## 2. Materials and Methods

### 2.1. Sample Preparation

CSF from TBI patients was collected at Pula General Hospital (Pula, Croatia) by placing ventriculostomy as part of their therapeutic intervention for intracranial pressure monitoring and management in the Intensive care unit. The CSF from 4 patients (three male patients of ages 24, 68 and 73 and one female, age 71) with no known comorbidities was pooled (Supplement Table S1). Specimens were collected in low-protein-binding polypropylene tubes (Eppendorf, Hamburg, Germany) and stored at –80 °C. All experiments were approved by the Ethic Committees of Pula General Hospital. Signed informed consent was obtained for all TBI patients. 

### 2.2. Size-Exclusion Chromatography

The gravity-driven SEC was adopted according to the previous optimization [24], but with an increased load volume. Five milliliters of pooled CSF from 4 TBI patients (CSF pool) was loaded to the glass SEC column 1.5 × 50 cm with flow adaptor and 30 µm bottom frit (Bio-Rad Laboratories, Hercules, CA, USA). The stationary phase was Sepharose CL-6B (GE Healthcare, Danderyd, Sweden) and the mobile phase phosphate buffered saline (PBS; Gibco, Thermo Fisher Scientific, Waltham, MA, USA). The column was first washed with distilled water and equilibrated in PBS; then, 35 fractions 2 mL in volume were collected in low-protein-binding tubes after 5 mL of void volume. The SEC was performed in duplicate.

### 2.3. Slot Blot, Western Blot, Immunoblot

Two-hundred microliters of a SEC fraction was added to 50 µL of 5× Laemmli buffer without glycerol (1M Tris HCl pH 6.8, 10% sodium dodecyl sulfate (SDS; *w*/*v*), 0.05% bromophenol blue (*w*/*v*), 2-mercaptoethanol) and boiled at 95 °C for 10 min. Slot blot was performed on a nitrocellulose membrane (GE Healthcare Life Science, Uppsala, Sweden) by applying slot blot apparatus (Hoefer Scientific Instruments, Holliston, MA, USA), and a volume of 250 µL was loaded per slot. Membranes were stained with Ponceau S (0.1% Ponceau S in 5% acetic acid), blocked with 5% milk in Tris-buffer saline (TBS; 20 mM Tris and 150 mM NaCl) for 15 min and incubated overnight at 4 °C on a shaker with rabbit monoclonal antibodies against CD81 (#56039) and CD9 (#13403) 1:1000 in 5% bovine serum albumin (Roche Diagnostics, Mannheim, Germany) prepared in TBS supplemented with 0.1% Tween 20 (BSA/TBS-T). Membranes were washed three times for 5 min in TBS-T and incubated with horseradish peroxidase-linked anti-rabbit (#7074) secondary antibody at room temperature for 30 min on a shaker. After additional washing in TBS-T, the signal was visualized through chemiluminescence of SignalFire Elite ECL Reagent (Cell Signaling Technology, Danvers, MA, USA) and imaged with imager ImageQuant LAS 4000 mini (GE Healthcare, Chicago, IL, USA).

Western blot was used for confirmation of unspecific binding of antibodies in the slot blot. The EV pool was created by mixing the first 3 fractions (6 mL) that gave a positive signal in CD9 and CD81 immunodetection slot blots, i.e., fractions 8–10; the protein pool was formed from 3 later eluates (6 mL) that gave strong signals in all three slot blot analyses (with Ponceau S staining, CD9 and CD81 immunodetection), i.e., fractions 23–25. For Western blot, 16 μL of CSF pool, 44 μL of EV pool and 44 µL of protein pool were boiled at 95 °C for 10 min with addition of 5× Laemmli buffer (1M Tris HCl pH 6.8, 50% glycerol (*v*/*v*), 10% SDS (*w*/*v*), 0.05% bromophenol blue (*w*/*v*), 2-mercaptoethanol) and electrophoresed on 15% SDS polyacrylamide gel electrophoresis (SDS-PAGE) (Bio-Rad) in 1× running buffer (25 mM Tris, 192 mM glycine and 0.1% SDS, pH 8.3) from 90 to 150 V. Proteins were transferred to 0.2 µm nitrocellulose membrane (GE Healthcare Life Science, Uppsala, Sweden) at the constant voltage of 70 V for 1.5 h. Membranes were stained with Ponceau S followed by blocking with 5% milk in TBS for 15 min and incubated overnight at 4 °C on a shaker with rabbit monoclonal antibodies against CD81 (#56039) and CD9 (#13174) 1:200 and albumin (#4929) 1:1000 in 5% BSA/TBS-T. Membranes were washed three times for 5 min in TBS-T and incubated with horseradish peroxidase-linked 1:1000 anti-rabbit (#7074) in 5% bovine serum albumin/TBS-T at room temperature for 30 min. After additional washing in TBS-T, signal was visualized using SignalFire Elite ECL Reagent (Cell Signaling Technology, Danvers, MA, USA) and imaged with imager ImageQuant LAS 4000 mini. All antibodies were purchased from Cell Signaling Technology.

### 2.4. Tunable Resistive Pulse Sensing

Tunable Resistive Pulse Sensing (TRPS) was applied for measuring the concentration, size distribution and zeta potential of particles in the EV pool by qNano Gold (Izon Science, Christchurch, New Zealand). Reagent kit, NP400 (185–1100 range) and NP150 (70–420 range) nanopores, carboxyl polystyrene particle standards (CPC100 and CPC400) were bought from Izon and all solutions were prepared according to the manufacturer’s instructions. Data collection and analysis were performed with Izon Control Suite 3.4 according to the manufacturer’s instructions. Measurement on NP150: pressures 2 and 4 mbar, stretch 49.01 mm, voltage 0.4 V, average current ~125 nA, average RMS noise ~15.0 pA, particle count: 500–550, particle rate ~230 particles/min. Total number of analyzed particles was 2331. NP400: pressures 3 and 6 mbar, stretch 49.5 mm, voltage 0.24 V, average current ~140 nA, average RMS noise ~17.0 pA, particle count: 500–550, particle rate ~300 particles/min. Total number of analyzed particles was 3267. Samples were diluted 1:1 with the measurement electrolyte provided in the Izon reagent kit. The instrument already took into account the dilution when giving out the data about the particle concentration. Zeta potential of nanoparticles was determined only on NP400 nanopores. Initial calibration with CPC400 was conducted at one pressure condition and 3 applied voltages, generating corresponding baseline currents of 90, 120, and 150 nA, followed by an additional measurement at the second pressure condition at the highest voltage applied in the previous step. Sample measurements were completed at the highest calibration voltage and pressure. Concentrations by TRPS were calculated as arithmetic means of four measurements for NP150 (two replicates for each pressure) and six measurements for NP400 (three replicates for each pressure).

### 2.5. Nanoparticle Tracking Analysis

Before NTA, absorbance was measured spectrophotometrically (BioTek Instruments Inc., Winooski, VT, USA) at 280 nm (A280) to evaluate the impurity content of each sample. Particle concentration and size in the EV pool were then determined by NTA using the NanoSight NS300 instrument (488 nm laser) connected to an automated sample assistant (both Malvern Panalytical, Malvern, UK). Samples were diluted 2 times in PBS and recorded five times at camera level 12 (sCMOS, Oxford Instruments, Abingdon, United Kingdom). Raw data were analyzed by NanoSight NTA 3.3 program at the following settings: detection threshold 5, water viscosity, temperature 25 °C, automatic settings for minimum expected particle size and blur and minimum track length 10. Total number of analyzed particles was 3.1 × 10^5^. Output data were expressed as EV concentrations, i.e., number of particles per 1 mL of EV pool (6 mL was the final volume of EV pool); EV size as a histogram with 5 nm bins and percentiles. The final concentration of EVs by NTA was calculated as double the value of the 2× diluted sample and the arithmetic mean from three sample replicates.

### 2.6. Atomic Force Microscopy

Muscovite mica, grade V-21, with 12 mm diameter (SPI Supplies, West Chester, PA, USA) was functionalized with 50 µL of 10 mM NiCl_2_ and dried under the nitrogen stream. Then, 100 µL of sample was placed on mica for 15 min and analyzed using Dimension Icon (Bruker, Billerica, MA, SAD) with ScanAsyst (Bruker) probe in liquid in tapping mode. The obtained images were analyzed with Gwyddion 2.60 (Czech Metrology Institute, Brno, Czech Republic) according to the procedure by Skliar and Chernyshev [25]. Maximum Martin diameters of particles were collected from four 10 µm × 10 µm images (see Supplementary Figure S1 as an example of particle recognition from an AFM image). A total of 669 particles was analyzed.

The crop of a single *EV* was performed on six 5 µm × 5 µm images, and a total of 433 EVs were analyzed to obtain the frequencies (%) of different morphologies.

Concentration (*c*) of *EVs* by *AFM* was calculated as arithmetic mean of values from the 4 images by considering the image sizes (10 µm × 10 µm), the sample volume (100 µL) and the total mica surface area (113.1 mm^2^) [26]:

$$c(EVs)=\frac{number\;of\;particles\;in\;the\;AFM\;image\;}{pipetted\;volume\;of\;EV\;pool\;}\times \frac{surface\;area\;of\;mica\;}{area\;of\;the\;AFM\;image}$$

### 2.7. Cryogenic Transmission Electron Microscopy

Samples for cryo-TEM were prepared as previously described [27]. An aliquot of 3 μL of the aqueous sample was applied on glow-discharged (30 s, 30 mA) lacey carbon EM grids (Micro to Nano, Haarlem, The Netherlands). The excess of the sample was blotted for 2.0 s and plunge frozen into liquid ethane held at −183 °C using Vitrobot Mark IV (FEI, Hillsboro, OR, USA). The samples were observed in a Transmission Electron Microscope (TEM) JEM-2100Plus (JEOL, Tokyo, Japan) equipped with LaB_6_ cathode and TVIPS XF416 CMOS camera (TVIPS GmbH, Gauting, Germany). The images were taken at 200 kV and beam current 106 µA under cryogenic conditions using Gatan 626 cryo-transfer holder and SerialEM software version 4.0.0beta (developed in Boulder Laboratory for Three-Dimensional Electron Microscopy of Cells, Department of Molecular, Cellular, and Developmental Biology, University of Colorado, Boulder, CO, USA) with the pixel size of 0.3777 nm (×30 k) and defocus target set to −3.0 µm. Twenty EV structures were recognized in the images and analyzed. 

### 2.8. Statistical Analysis

The measured particle diameters were sorted by size to obtain cumulative distributions. Then, a log-normal cumulative distribution function was fitted to the data using Gnuplot software and the mean, mode, median and variance of the size distribution were extracted from the fit parameters. Coefficient of variation (CV) was calculated as the standard deviation σ (square root of variance) of the fitted distribution, divided by the mean value. For NTA, TRPS NP150 and TRPS NP400, fitting was conducted on each sample replicate (3 for NTA, 4 for TRPS NP150, 6 for TRPS NP400); and arithmetic means and standard deviations were calculated from the replicate data fits. For AFM and cryo-TEM, measurements from all analyzed images were taken together for cumulative distribution fitting. Percentile values (d10, d50, d90 and span = (d90–d10)/d50) were calculated from raw data, except for cryo-TEM, for which they were rather calculated from the log-normal curve due to small number of measurements. TRPS combined data were constructed from the TRPS NP150 and TRPS NP400 data by weighing each measurement with its number fraction and the concentration of its corresponding sample replicate, then averaging over all NP150 and NP400 replicates for every size. For visual representation of size distributions, the measured particle diameters were bucketed into histograms with bins 30 nm in size (50 nm for cryo-TEM). The numbers/concentrations of particles were divided by the total number/concentration to obtain frequencies. Log-normal probability density functions with parameters from the cumulative distribution fits were plotted over the corresponding histograms. For NTA, TRPS NP150 and TRPS NP400, histograms were calculated for each replicate, and then the arithmetic mean and standard deviation were placed in each bin. The results with standard deviations were compared for statistical significance using a two-tailed Student’s/Welch’s *t*-test with exact *p* values provided. Zeta potential from TRPS NP400 was obtained from a 2D histogram over sizes and charges (3D plots); then, the average mode from two measurements was used.

## 3. Results

### 3.1. Identification of EVs in the SEC Eluates through the Analysis of Their Protein Content

CSF was pooled from four TBI patients, whose Glasgow coma scale (GCS) score at admission and discharge and Glasgow outcome scale (GOS) score three months after discharge are shown in Supplementary Table S1. EVs were isolated from the CSF pool using gravity-driven SEC according to the protocols described previously [24]. In total, 35 fractions of 2 mL each were collected from the 5 mL CSF pool after SEC, and the slot blot was performed. 

**Figure 1:**
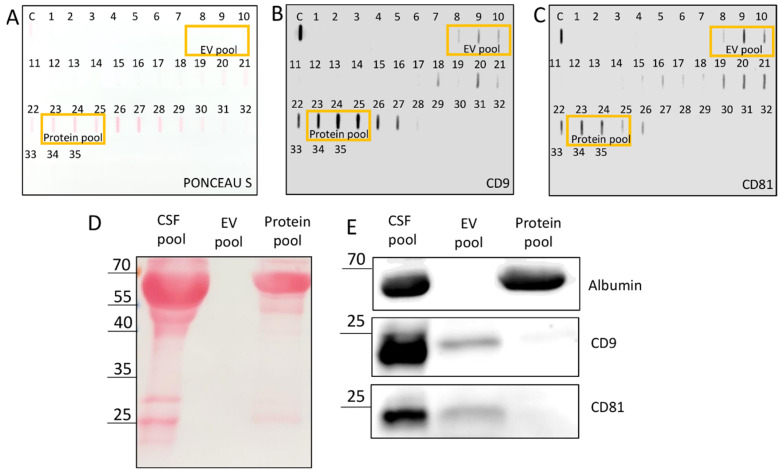
Tetraspanins CD9 and CD81 were present in the EV pool. Slot blot of the CSF pool and the eluates in the gravity-driven size-exclusion chromatography (SEC), with Ponceau S staining (**A**) and immunodetection based on CD9 (**B**) and CD81 (**C**); the fractions that composed the EV pool and the protein pool are marked with rectangles. Ponceau S staining of CSF pool, EV pool and protein pool separated by SDS-PAGE (**D**) and Western blot on albumin, CD9 and CD81 of CSF pool, EV pool and protein pool. Sizes of detected proteins are indicated in kilodaltons, kDa (**E**). CSF: cerebrospinal fluid; C: cerebrospinal fluid pool; EV: extracellular vesicle; SDS: sodium dodecyl sulfate; PAGE: polyacrylamide gel electrophoresis.

Ponceau S staining of slot blot revealed the presence of total proteins in the CSF pool and enrichment in fractions 22–30 (Figure 1A); and slot blot with immunodetection confirmed the presence of tetraspanins CD9 and CD81 in fractions 8–10 and ~15–28 (Figure 1B,C). To check the specificity of the tetraspanin protein chemiluminescence signal on the slot blot, fractions 8–10 were mixed into an EV pool (6 mL) and late eluting fractions 23–25 (with signal in both the slot blot with Ponceau S staining and the slot blot with tetraspanin immunodetection) into a protein pool (6 mL). Western blot was then performed on a 1.5 mm thick 15% SDS gel, with increased load volumes for the EV pool and protein pool (16 μL load volume for CSF pool but 44 μL for EV pool and protein pool) to account for the dilution during SEC. Ponceu S staining of the membrane after electrophoresis is shown in Figure 1D. The signal of the CSF pool separated into several different proteins. The strongest line was for the protein below 70 kDa, and other visible lines were at around 55 kDa and between 20 and 35 kDa. The line below 70 kDa could be distinguished also for the protein pool, along with a line above 25 kDa, but the signal for the EV pool was extremely weak. The three samples were further compared by content of specific proteins (albumin, CD9 and CD81) through immunoblot (Figure 1E). Albumin was identified as the protein below 70 kDa, and its presence was confirmed for the CSF pool and the protein pool but not for the EV pool. On the other hand, CD9 and CD81 (identified as proteins with molecular masses below 25 kDa) were confirmed for the CSF pool and the EV pool but not for the protein pool. 

### 3.2. Quantification of EVs by NTA, TRPS, AFM and Cryo-TEM

**Figure 2:**
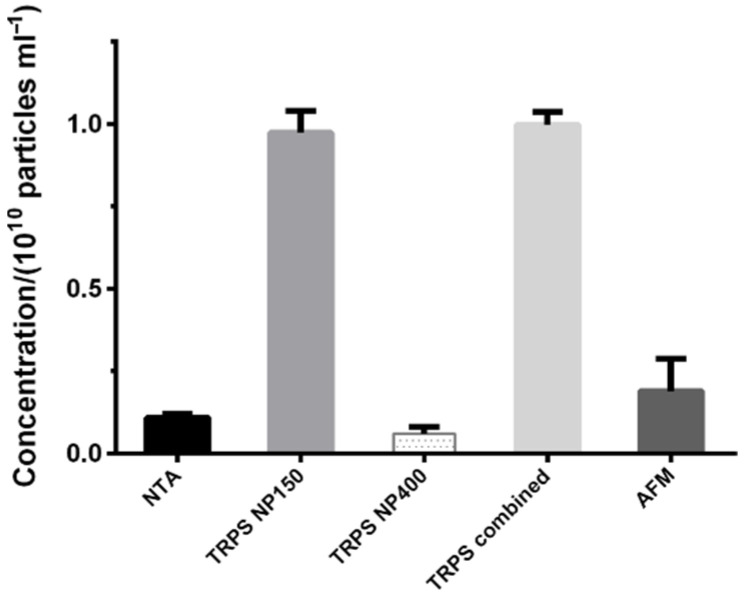
Concentrations in the EV pool obtained by Nanoparticle Tracking Analysis (NTA) and Tunable Resistive Pulse Sensing (TRPS) measured with nanopores NP150 and NP400 and combined, together with Atomic Force Microscopy (AFM).

After identifying the EV-enriched SEC fractions and confirming their separation from free proteins (such as albumin), we continued to quantify their contents of particles. The particle concentrations measured by NTA and TRPS on two nanopores (NP150 and NP400) and AFM are compared in Figure 2. To evaluate the applicability and the performances of different EV quantification methods, we compared the results obtained by two commonly applied methods, NTA and TRPS, with the data obtained by AFM and cryo-TEM. The measured size range, mode, mean, median, d90 and d10, span, CV, distribution fit data and volume needed for a measurement are presented in Table 1.

TRPS with nanopore NP150 detected 9.7 ± 0.7 × 10^9^ particles/mL in the EV pool, which is significantly higher than the concentrations found by the other methods (*p* = 7 × 10^−5^ with respect to NTA and *p* = 3 × 10^−5^ with respect to AFM) and more than 15 times higher than the concentration detected by TRPS with nanopore NP400 (*p* = 4 × 10^−5^). This indicates that there was a substantial population of particles below 160 nm in the EV pool that was cut off by the NP400 filter. This was further confirmed by its narrower size distribution (the lowest span and CV) and the fact that its combination with the NP150 filter did not alter the mode size (Table 1). 

AFM and NTA detected a similar concentration of particles (*p* = 0.20), but their most frequent size was evidently larger in NTA (151 vs. 61 nm). TRPS NP150 determined an in-between value of around 100 nm. The mode by NP400 was significantly larger (above 240 nm) than by other methods (*p* < 10^−5^ with respect to TRPS NP150 and NTA) but its detection limit was already above all other measured mode diameters. Cryo-TEM distribution was based only on 20 particles, so it should be considered with caution. Nevertheless, its mode size was close to the ones of the TRPS NP150 and the TRPS combined size distributions, and its CV and span values were comparable to the ones obtained by other methods. 

**Figure 3:**
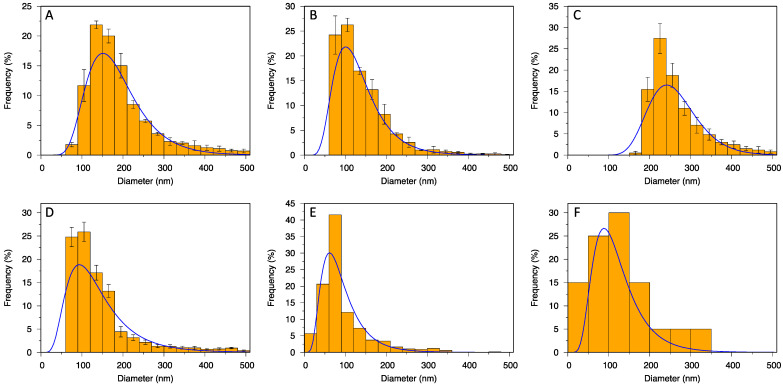
Size distribution histograms of EV pool obtained by NTA (**A**); TRPS with NP150 (**B**), with NP400 (**C**) and combined (**D**); AFM in liquid (**E**); and cryo-TEM (**F**).

The data from the cumulative distributions were also visualized in histogram frequency distributions (Figure 3), which confirmed adequate goodness of cumulative fits and unimodal particle size distribution. This was true even for TRPS combined (Figure 3D), which indicates that the mode above 240 nm obtained by TRPS with NP400 (Figure 3C) was not an evident second mode in the overall particle size distribution. TRPS measured size in a predefined range of the nanopore, which resulted in a cut-off in the histogram and the interruption of the left parts of the fitting curves, below 60 nm for NP150 (Figure 3B) and below 160 nm for NP400 (Figure 3C). Even though the results by different methods differed in the detected size range, they all encompassed the most important part of the particle size distribution, apart from TRPS with NP400. Particles with diameters above 300 nm were extremely rare and contributed very little (Figure 3), 90% or more particles were smaller than 330 nm (d90 values in Table 1) and only 10% or less were smaller than 40 nm (d10 values in Table 1). Only NTA and TRPS with NP400 found the particles larger than 350 nm at a higher frequency. Taking all the results together, the EV pool contained particles spanning mainly from 40 to 260 nm, most of which were about 60–100 nm in size and around 150 nm in hydrodynamic diameter.

Zeta potential was measured with TRPS using nanopore NP400, which yielded 30 ± 3 mV (Figure S2). With nanopore NP150, we could not measure zeta potential due to clogging of the particles on the nanopore.

### 3.3. Native Morphology of EVs by AFM in a Liquid Environment and Cryo-TEM

**Figure 4:**
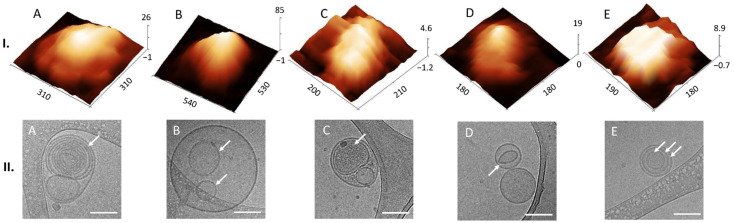
Near-native morphologies of EVs with preserved lumens. 3D AFM images of particles in the EV pool (**row I**) with different shapes and structures: multilobed (**A**), round (**B**), elongated bulging (**C**), single-lobed flat (**D**) and flat (**E**); dimensions in nm. Cryo-TEM images of different EV structures present in the EV pool (**row II**) with different structures marked with white arrows: onion-like internal structures (**A**), one or more vesicle inside one EV (**B**), which could have an electron-dense lumen (**C**), a deformed inner vesicle (**D**), two or more membranes (**E**). Bar 100 nm.

**Figure 5:**
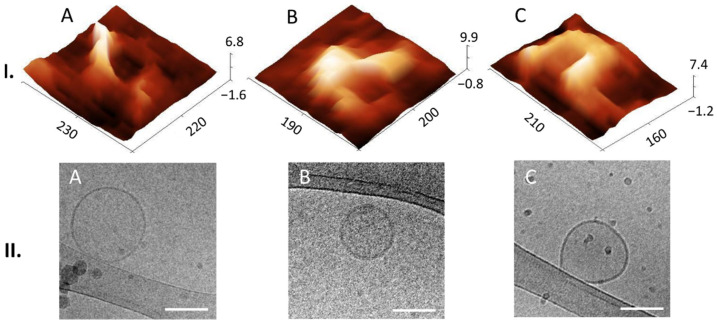
Near-native morphology of EVs with a collapsed lumen present in the EV pool. 3D AFM images of EVs (**row I**) with different cup-shaped structures (**A**,**B**), and partially open cup-shaped (**C**); dimensions in nm. Cryo-TEM images of different EVs (**row II**) with a single membrane bilayer (**A**); some appeared with an electron-dense lumen (**B**) or with damaged membrane (**C**). Bar 100 nm.

To investigate the native morphology of the quantified particles in the EV pool, we applied AFM tapping mode in liquid and obtained the images from the height signal. The particles were attached to a freshly cleaved mica surface with a positive charge (obtained by applying Ni^2+^ from a NiCl_2_ solution onto it) without adding any fixatives. Topographic 10 × 10 µm images showed no aggregation or disruption of the particles (Supplementary Figure S1). For detailed insight into the single EV shape and structure, we examined in detail six topographic images 5 × 5 µm in size, and the frequencies (%) of different morphology structures were calculated. 3D images of individual EV-like particles revealed different shapes of preserved lumen (Figure 4I) and collapsed lumen, so-called cup-shaped (Figure 5I). The structures with preserved lumen were mostly round or slightly elongated with distinct features, which we designated as multilobed (Figure 4(I.A)), round (I.B), elongated bulging (I.C), single-lobed flat (I.D) and flat (I.E). The multilobed structures could be round, with three or more lobes, and the elongated multiple-bulge structures could be visualized by changing the contrast. Some EVs appeared with a single-lobed substructure on a flat surface (Figure 4(I.D)), and some had a totally flat surface (I.E). The round structures exhibited a very smooth surface (Figure 4(I.B)). The cup-shaped EVs also appeared partially open (Figure 5(I.C)). Of the 433 individual EVs analyzed, 408 had a preserved lumen (17% multilobed; 33% round; 10% elongated bulging; 9% single-lobed and 31% flat) and 25 a collapsed lumen (64% cup-shaped and 36% partially open).

We visualized heterogenous subpopulations of EVs in near-native morphology also by cryo-TEM as multimembrane, which could support in-tact lumen (Figure 4II) and single-membrane (Figure 5II) morphology, which could lead to cup-shaped morphology. Different structures with membrane bilayers and different internal structures are presented and marked with white arrows in Figure 4II. We designated them as multimembrane onion-like internal structures (Figure 4(II.A)), one or more vesicles inside one EV (Figure 4(II.A–C)) and two or more membranes in a single EV (Figure 4(II.A,E)). EVs with electron-dense inner vesicles were also observed (marked with a white arrow in Figure 4(II.C)). Another structural variant was also found containing an elongated and deformed inner vesicle within an EV (marked with white arrow in Figure 4(II.D)).

Among the single-membrane EVs shown in the second row of Figure 5, some were damaged (Figure 5(II.C)) and a few exhibited more electron-dense lumens (Figure 5(II.B)). Cryo-TEM images contained also many darker particles with different darkness intensities and without a visible membrane bilayer, which were considered as artefacts and attributed to ice contamination (see Supplementary Figure S3). Of the 20 individual EVs analyzed, 60% were multimembrane and 40% were single-membrane.

## 4. Discussion

We separated EVs from other components of human CSF after TBI by gravity-driven SEC as the mildest isolation method, characterized their near-native morphology by applying AFM in liquid and cryo-TEM and quantified their concentration and size distribution by NTA, TRPS and AFM. Such characterization is in accordance with the Minimal Information for Studies of Extracellular Vesicles (MISEV) requirements [20] and crucial for any further investigations of physiological and pathophysiological changes of CSF and the brain based on the properties of EVs. 

SEC dilutes the sample, which can render the concentration of specific proteins in the eluates too much for their detection by chemiluminescence. To get a higher protein concentration in the eluates, we increased the load volume of CSF pool on the SEC column from 2.8 to 5 mL compared to our earlier publication [24]. Aiming to obtain an EV pool for further investigation, we performed slot blot on all SEC fractions using both total protein staining and specific immunodetection based on transmembrane CD9 and CD81 proteins, which span the phospholipid bilayer and are known markers also for EVs from CSF [6]. Since there was a positive chemiluminescence signal for these proteins, not only for the expected early fractions 8–10, but also for late eluting fractions, we separated the proteins by electrophoresis (Figure 1D,E) and compared the CSF pool with the EV pool (pooled fractions 8–10) and the protein pool (pooled late eluting fractions 23–25, which exhibited also visible Ponceau S staining). This confirmed that most of the CD9 and CD81 signals in the protein pool fractions came from unspecific binding of antibodies to free proteins, whereas they were most probably derived from EVs in the case of EV pool fractions. We could obtain positive signals of CD9 and CD81 on Western blot only after increasing the load volume to 44 µL. The dilution effect of SEC could be further optimized, possibly by combining different EV isolation methods [13,28]. Additionally, abundant soluble proteins, such as albumin, can give false positive results and should be eliminated from an EV sample by an appropriate isolation method. We confirmed the absence of impurities in the EV pool by zero spectrophotometrical readings of absorbance at 280 nm (A280) before NTA analysis. 

Unlike methods for biochemical determination (slot blot, Western blot), the SEC dilution factor did not limit the methods for particle quantification (NTA and TRPS), as they required even further dilution of the samples. Instead, there was a broad range of particle sizes in line with the method limits of detection. Nanopores were produced with defined measurement ranges (e.g., 70–420 for NP150; 185–1100 for NP400), which cuts the size distribution of particles. Thus, no particles below 60 nm could be counted in NP150, and no particles below 160 nm in NP400. A suggestion to deal with this issue would be to apply at least two nanopores and combine the obtained measurements (Table 1, TRPS combined, Figure 3D). Filtering particles above the top size limit of each nanopore could then prevent clogging, but it would also lead to the loss of particles. On the other hand, NTA detection is limited down to a certain size connected with the refractive index of the particles, which leads to diameters as low as 30 nm for EVs [15] (40 nm in our case). Moreover, NTA measures hydrodynamic rather than true particle diameter. It also has some limitations in polydisperse systems, causing inaccurate measurements of smaller particles [16], and dilution has to be finely tuned, which can be a problem in clinical samples [14]. Sample concentration can influence the measurements (for our setup we first measured 1000 µL and then a 2× diluted sample, since the concentration was too high for an accurate measurement). 

Liquid AFM analysis was also not much affected by the SEC dilution as the positive charge on the surface of mica enabled sufficient capture of EVs. However, the wide size distribution of particles in the sample caused a low detection limit, as larger images were required for analysis, so particles below ~30 nm were neglected. Nevertheless, cryo-TEM revealed that EV size went only down to around 20 nm; particles below 30 nm were mostly artefacts of frozen PBS. Thus, neither NTA nor AFM lost any important fraction of particles from the sample during the analysis. Cryo-TEM was affected by the diluted sample and part of the sample also got lost during the preparation procedure, so the number of particles was too low for reliable size-distribution statistics. Each method has its pros and cons, so it is suggested to combine and compare the results obtained by more than one method based on different working principles [16,19].

TRPS detected a higher concentration of particles than NTA and AFM. This could be explained by the higher degree of agglomeration in NTA (no use of surfactant as in TRPS) and its lower sensitivity to smaller particles in polydisperse samples, whereas in AFM the lower concentration might be related to a proportion of non-attached EVs, which then could not be observed and counted. 

If we compare the concentrations of EVs obtained by NTA and TRPS with the usual values when using the most adopted UC method for isolation, they are expectedly lower due to the dilution effect in SEC [17,29]. Nevertheless, the concentration of particles in the EV pool in this study measured by TRPS with NP400 nanopore ((6 ± 2) × 10^8^ particles/mL) is in agreement with our previous results using the same quantification method for a fraction positive for EVs obtained after SEC of CSF (6.02 × 10^8^ particles/mL) [24]. Even though it may seem that NP150 is a better choice for the size range of EVs than NP400, we should point out that the analysis by NP150 was extremely difficult due to clogging of the nanopore. For the same reason, we could only get the readings of zeta potential on NP400 (–30 ± 3 mV, Supplementary Figure S2). We used a native sample with no additional steps prior to placing it at −80 °C, which resulted in a broader range of particle sizes. 

NTA and TRPS cannot differentiate between EVs and other particles. Thus, Akers et al. [17] suggested that between 1/2 and 2/3 of particles measured with NTA and TRPS are protein aggregates and non-membranous particles. Nevertheless, EVs in that study were isolated by UC, in which protein aggregates co-isolate with EVs, contrary to SEC, which removes most of the protein impurities. Therefore, the latter were expected to contribute less to the total particle count by NTA and TRPS in our case.

Here we should also point out that we observed deterioration of EVs in PBS after chromatographical isolation, even when they were slowly frozen and stored at –80 °C (data not shown). We observed that the quantification readings for a sample at the same conditions could not be repeated after 3–6 months. This is important when planning the whole investigation that includes comparison of quantification methods and it should be minimized, e.g., by introducing rapid cooling with liquid nitrogen before storage at −80 °C. It has been shown that slow freezing may cause breakage of microparticles derived from human cell lines [30]. 

AFM has been rarely used as EV quantification method. In the present study, we obtained good agreement between AFM and NTA in particle concentration, but unlike in the work by Gazze et al. [23], the two methods did not yield similar mode particle diameters (around 60 nm with AFM vs. 150 nm with NTA). A sound explanation for this is that NTA measures hydrodynamic diameter and AFM solid particle diameter. However, the mode size by AFM was also notably smaller than by TRPS (around 100 nm) and cryo-TEM (around 90 nm), which both also measure true size. This might be the result of measuring maximum Martin diameters of 2D projections and overlapping of particle bases in AFM. Moreover, the size and shape of EVs are influenced also by their interactions with the tip and the functionalized substrate [23]; and with the size distribution obtained from large images at low magnification, without examining each counted particle in detail (as in cryo-TEM), there is also a possibility of counting non-EV precipitates/artefacts. Nevertheless, the mode size 60–100 nm agrees well with the mode size of EVs in the work by Emelyanov et al. [29]. Moreover, they also identified most of the EVs below 100 nm as single-membrane. The observed size range of 40–260 nm is also in agreement with previous studies of EVs from CSF, even though most of them were measured by conventional TEM in air-dried samples [31]. Conventional TEM also usually identified only concave (so-called “cup-shaped”) particles due to damage during air-drying, and the addition of heavy metals led to artefacts. Cryo-TEM avoids these issues and reveals the morphology much closer to the native in liquid, as the preparation protocols for cryo-TEM quickly vitrify the sample, which thus remains hydrated during imaging, while water crystal formation inside the EVs is prevented [32]. However, small water and liquid crystals can form around the particles and can be mistaken for particles from the sample but not for EVs, which possess clearly evident membranes. The absence of contrast agents requires the use of holey TEM grids, which might result in higher loss of EVs for observation and consequently hinder their statistical evaluation.

Liquid AFM and cryo-TEM revealed various morphological features of EVs. In AFM we could find multilobed, round, elongated-bulging, single-lobed flat, flat and concave (cup-shaped) EV structures. EVs imaged by cryo-TEM were identified by onion-like multimembrane internal structures, one or more vesicle being inside one EV, two or more membranes being in a single EV, EVs having electron-dense inner vesicles and elongated and deformed inner vesicles being within EVs. Images of EVs from CSF obtained by AFM are scarce, and this publication shows details of 3D morphology of single EVs. 

Emelyanov et al. [29] classified human EVs isolated by UC from CSF of Parkinson`s disease patients and patients with neurosurgical pathology by the presence of a lipid bilayer/membrane. They observed single, double and multimembrane vesicles, as we did in our analysis. Busatto et al. [33] also found similar multimembrane structures of EVs derived from regular human MDA-MB-231 breast cancer cells and a brain metastases variant of MDA-MB-231 cells. Peruzzotti-Jametti et al. [34] observed by cryo-TEM that EVs from C57BL/6 mice’s neural stem cells can even carry intact mitochondria with conserved membrane potential and respiration confirmed by functional analyses. Gallard-Palau et al. [35] compared different methods of EV isolation and suggested that multilayer structures are a consequence of UC. Since this method for isolation was used also in the study by Emelyanov et al., they still questioned whether such structures were in fact the result of the centrifugation. However, the results of the present study disprove this hypothesis, since we found the same kind of structures also among the EVs isolated by SEC. Nevertheless, we allow the possibility that the use of UC would create additional multilayer particles from natively single-membrane EVs. It has been shown previously that UC causes morphology changes and some structural bilipid damage [36].

To our knowledge, this study is the first to identify different 3D structures of EVs from CSF using AFM in a liquid environment. Moreover, we were able to connect these structures to the 2D internal morphologies observed in the cryo-TEM images. Thus, we could observe that membranes can support the structural integrity of the lumen of EVs, since they possess a distinct elastic, trilobed structure [37]. By comparing the AFM images of different EV 3D structures (Figure 4I) with the cryo-TEM images of different EV internal structures (Figure 4II), we can draw a conclusion that onion-like internal structures (Figure 4(II.A)), with one or more vesicles inside one EV (Figure 4(II.B)) or with two or more membranes (Figure 4(II.E)), could support the lumen and prevent its collapse, which has been shown also by measurements of nanomechanical properties [38]. On the other hand, if only one membrane is present with no internal membranous structures, the lumen could collapse and form a cup shape (comparison of AFM and cryo-TEM images in Figure 5). We have observed EVs with cup-shaped structure in liquid AFM before (Figure 5D of [22]). Possible reasons for their formation could also be their strong attachment to the functionalized mica or interaction with the AFM tip [23]. However, these structures are very rare in a liquid sample; they become much more abundant if drying is included in the sample preparation (Figure 5A of [22]). It is not known whether specific internal, multimembrane EV structures are caused by their biogenesis, or if they can be the results of particular physiological or pathophysiological conditions [39,40]. 

## 5. Conclusions

There is a lack of unambiguous identification and quantification of different subpopulations of EVs that exist in human CSF based on their morphology, which is necessary for any application in prognostics/diagnostics and liquid biopsy. Here we demonstrated the usefulness of combining different methods in unveiling the various types of EVs that are present in the CSF. To our knowledge, this work is the first to combine AFM in liquid for surface morphology investigation and cryo-TEM for internal structure differentiation of EVs from CSF. We believe that this systematic approach with combined methodology could lead to more reliable correlations in future investigations of the disease contributions of the properties of EVs. Moreover, the obtained results could importantly contribute to comprehensive understanding of the observed EV subpopulations in the human CSF. To correlate the different EV morphologies to their biological functions, further research should integrate other methods of microscopy and nanotechnology. 

## Figures and Tables

**Table 1:** Quantification data (based on log-normal non-linear fits of cumulative size distributions) for EV pool obtained by NTA and TRPS measured with nanopores NP150 and NP400 and combined, together with AFM and cryogenic electron microscopy (cryo-TEM) data. Errors for NTA and TRPS data are standard deviations of the arithmetic mean of sample replicates.

	NTA	TRPS NP150	TRPS NP400	TRPS Combined	AFM	Cryo-TEM
Size range (nm)	40–985	60–546	161–1319	60–1319	29–456	21–328
Mode size (nm)	151 ± 5	101 ± 4	241 ± 7	94	61	88
Mean size (nm)	187 ± 2	133 ± 6	262 ± 11	139	91	121
Median size (nm)	174 ± 3	121 ± 5	255 ± 9	122	79	109
d10; d90 (nm)	114 ± 2; 324 ± 9	78 ± 2; 219 ± 9	202 ± 4; 416 ± 32	77; 258	43; 172	60; 196
Span ((d90–d10)/d50)	1.23 ± 0.05	1.17 ± 0.06	0.9 ± 0.1	1.53	1.55	1.24
Coefficient of variation	0.39 ± 0.02	0.45 ± 0.01	0.24 ± 0.02	0.55	0.55	0.48
Number of analyzed particles	1.0 ± 0.4 × 10^5^	583 ± 64	544 ± 21	5598	669	20
Sample volume (µL)	500	17.5	17.5	17.5	100	3
Dilution for measurement	2×	2×	2×	2×	no	no

## Data Availability

The data presented in this study are available on request from the corresponding author.

## Supplementary Materials

The following are available online at https://www.mdpi.com/article/10.3390/biomedicines10061251/s1. Table S1: Description of severe traumatic brain injury patients included in the study with Glasgow coma scale (GCS) score at admission and discharge and Glasgow outcome scale (GOS) score three months after discharge. Figure S1: Representative AFM image 10 × 10 µm applied for size distribution analysis of EV pool. Figure S2: Zeta potential of EV pool obtained by TRPS with nanopore NP400 with marked value. Figure S3: Possible artefacts obtained by cryo-TEM, which are attributed to ice contamination.
