# Supplementary material for: Unveiling the Native Morphology of Extracellular Vesicles from Human Cerebrospinal Fluid by Atomic Force and Cryogenic Electron Microscopy
Source: Biomedicines. 2022 May 27;10(6):1251. doi: 10.3390/biomedicines10061251 (PMC9220600; doi:10.3390/biomedicines10061251)
Supplement: Supplementary file 1 [file biomedicines-10-01251-s001.zip › biomedicines-1700718-supplementary.pdf]

Table S1: Description of severe traumatic brain injury patients included in the study with Glasgow coma scale (GCS) score at admission and discharge and Glasgow outcome scale (GOS) score three months after discharge

**Patient**	**Age**	**Gender**	**Mechanism of injury**	**GCS at admission**	**GCS at discharge**	**GOS three months after discharge**	**Intracranial pathology**
1	73	M	motor vehicle accident	3	15	3	diffuse axonal injury (DAI)
2	71	F	fall	3	exitus letalis	1	subdural haematoma (SDH), subarachnoid hemorrhage (SAH), intracerebral haematoma (ICH)
3	68	M	fall from height	6	exitus letalis	1	SAH
4	24	M	motor vehicle accident	5	15	5	DAI

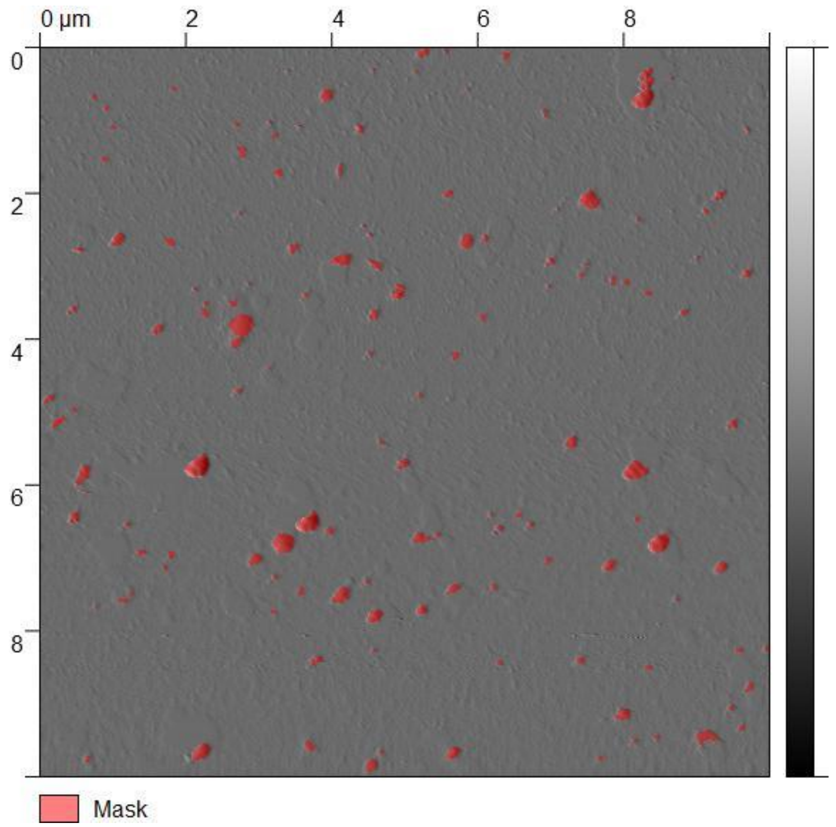

Figure S1: Representative AFM image 10 x 10 µm applied for size distribution analysis of EV pool

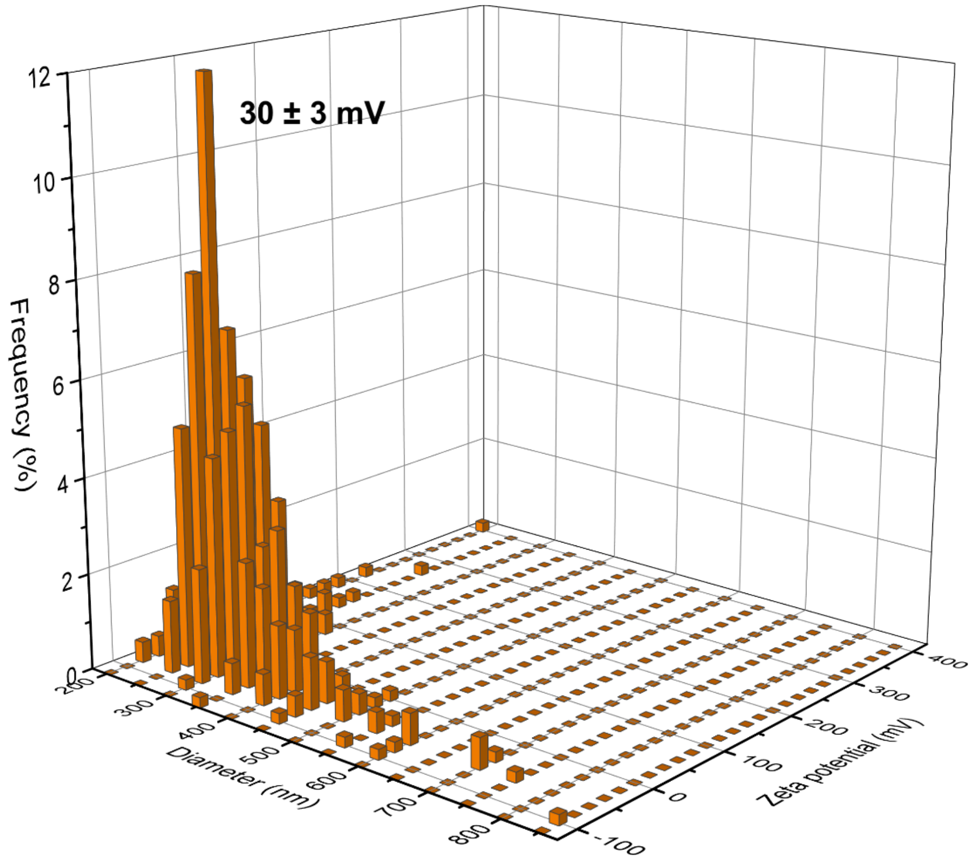

Figure S2: Zeta potential of EV pool obtained by TRPS with nanopore NP400 with marked value

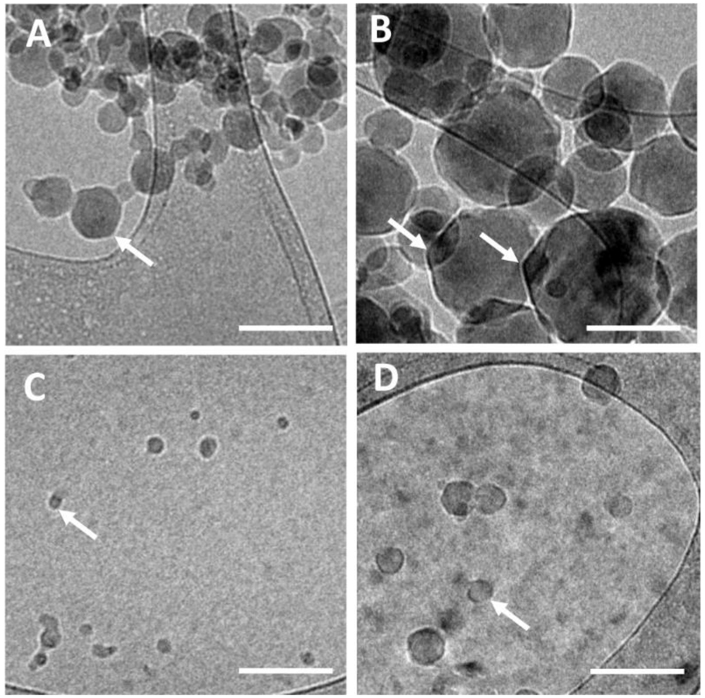

Figure S3: Possible artefacts obtained by cryo-TEM, which are attributed to ice contamination
